# Hypnotism in Therapeutics

**Published:** 1889-11

**Authors:** 


### HYPNOTISM IN THERAPEUTICS.

This seductive method of treatment is slill on the increase. Its exponents are for the most part more enthusiastic than ever. The therapeutical practice of hypnotism is no longer circumscribed by the narrow confines of France. It has spread throughout Continental Europe. Its progress has not been stayed by that watery Gibraltar, the English Channel, but sailing across has laid hold upon the insular conservatism of Great Britain. It has reached Germany, Sweden and Russia. It has scaled the Alps from all sides and well nigh taken Switzerland by storm. It is more popular in staid Holland than in France. America alone has thus far escaped—but we expect it soon as we do all widespread contagion. Let us see, then, to what we may look foward. Hypnotism, not long since wrested from the hands of charlatanism, is held as a trophy by the medical profession. What we know of it in this country is mainly what we have learned of its employment by Charcot in the Saltpetriere. At present, however, there are in France two schools of hypnotism—that of Nancy, with Bernheim, Liebault and Liegois as its principal champions, and that of Paris, where its practice has been developed under the influence of Charcot. The former school is by far the more radical, and its teachers claim the wider field of applicability for the method.

Nearly thirty years ago Dr. Liebault began to treat dispensary patients in Nancy by a system which he elaborated and termed "Treatment by Suggestion." In 1866 he published a book in which he gave a full description of his various methods, together with a report of cases successfully treated. Nevertheless, he remained for many years a prophet without honor. In 1844 Prof. Bernheim of Nancy, although skeptically inclined, began an investigation of the subject with the result of soon becoming a convert, and in 1884 he published his classical work, a second edition of which has recently appeared in this country, bearing the title, "Suggestive Therapeutics: a Treatise on the Nature and Uses of Hypnotism." Hitherto the English language has been practically without a literature on this subject. To Liebault is due the credit of having founded a school of practice which has found representatives throughout Europe. The interest in the entire subject which has been gradually increasing, and which has been fostered by discussions in the medical societies of various European cities has finally culminated in a "Congress of Hypnotism" held at Paris, where congresses of all possible descriptions have been unprecedentedly numerous of late. At this Congress the forces were marshalled under the leadership of the aforesaid schools of practice. The Nancy contingent, with its radical views, was well equipped and ably generaled by Prof. Bernheim, whose paper on the "Therapeutical Value of and the means of Inducing Hypnotism" served as a point of departure for a pretty lively dispute.

The principal differences between the two schools may be briefly explained. In the first place, the Nancy school believes that hypnotism is a physiological condition; the Paris school insists that it is a neurosis. The Nancy school thinks that pretty much all the manifestations of hypnotism are produced by suggestion and that the hypnotic state is so produced. The Paris school complains that hypnotism and suggestion are two separate things, and that Prof. Bernheim is so absorbed in suggestion that he has lost sight of hypnotism altogether.

These are some of the victories claimed by the champion of Nancy: Nearly all the tedious and rebellious cases of hysteria, hyperæsthesia and amblyopia almost instantly relieved; pains from lead poisoning, neuralgia and rheumatism instantaneously or very quickly subdued; chronic rheumatic athritis cured; pleurisy pains removed in one or several sittings; chorea quickly cured or greatly shortened in its duration; menstruation regulated, checked or made to appear at a fixed date; spinal disease ameliorated; an obstinate case of vomiting of long standing cured in two weeks; a case of cerebro-spinal sclerosis with palsy and staggering relieved for a long period; an old and most obstinate case of migraine with insomnia cured in three weeks, etc.

Charcot, however, holds very different opinions. Without wishing to deny that in cases of organic disease of the nervous system hypnotic suggestion may, in certain cases, result in a degree of improvement, he is convinced that this occurs only by mere accident, and that, in such cases, the methods of suggestion have no claim to be regarded as therapeutical measures. On the other hand, in cases of hysteria in women and individuals markedly susceptible to hypnotism in its stage of somnambulism, good results may reasonably be expected. As for hysteria in men, one must express one's self with greater reserve and beware of insisting upon a method which is very far from giving good results in all cases and may, indeed, produce exactly opposite effects with very disagreeable consequences. One should deal with hypnotism as with all other therapeutical measures; it has its indications and its contra-indications and if one fails to employ it in a judicious manner the results are apt to be disastrous. Charcot's application of hypnotism has been confined to neurotic patients, and with them he seems of late to be somewhat less enthusiastic than formerly.

It is, however, from the Nancy school that we receive the most glowing accounts of cures by hypnotism. As for the other side of the question, we hear but little from the opponents of hypnotism in France, where, if the subject is mentioned at all, it is spoken of in terms of praise.

In England a variety of opinion is expressed, much of which is favorable to the method. In connection with a paper read at the recent meeting of the British Medical Society by Dr. Voisin, of Paris, several English physicians reported successful cases, while others admitted having received a favorable impression by witnessing its action in England and France. Dr. C. L. Tuckey has also written an interesting brochure, entitled "Psycho-Therapeutics," a large part of which is taken up with an ingenious argument of inductive reasoning with a view to explaining the phenomena of hypnotism.

In Germany, although there is a growing interest manifested in the subject, there is not wanting an expression of wholesome caution and distrust, as indicated by recent expressions from writers of eminence, some of whom do not hesitate to pronounce hypnotism a very dangerous agent. Among those in Germany who are very decidedly opposed to hypnotism may be mentioned Prof. von Ziemssen, who, as the result of a series of experiments conducted by his assistants, has reached the conclusion that hypnotism exerts little or no beneficial action even in cases of slight functional disturbance, while it is positively injurious to many patients. He believes that to employ the method as a therapeutical measure in cases of slight functional disturbance is like gunning for sparrows with cannon balls for ammunition, while its repeated application is apt to convert the milder forms of hysterical manifestations into the *granude hysterie* of Charcot; in the severer forms of diseases it is a mere substitution of one form of mental disorder for another, and is akin to curing one of the morphine habit by giving him cocaine. He trusts that the good sense of the medical profession in Germany will restrain it from countenancing so dangerous a procedure.

There can be no doubt that the elements of faith and expectancy are prime factors in the solution of this problem, just as they are in the somewhat allied methods of faith cure, mind cure, prayer cure, and, may we not add, in classical homœopathy as well. Expectancy of cure in the mind of the patient is one of the most potent allies of therapeutics, and one which the physician ought never to disregard or underestimate. As for hypnotism itself, it will be well for all who are inclined to experiment with it to remember the failures and dangers that have already been encountered, and not blindly trust the voice of mere enthusiasm.—*The Journal.*

